# ACCREDITATION FOR GERONTOLOGY EDUCATION COUNCIL: OVERVIEW AND EXPERIENCES TO DATE

**DOI:** 10.1093/geroni/igz038.1469

**Published:** 2019-11-08

**Authors:** Harvey L Sterns

**Affiliations:** The University of Akron, Akron, Ohio, United States

## Abstract

This presentation will describe the accreditation process that was developed by a special committee under the sponsorship of the Association for Gerontology in Higher Education. With support from The Russell & Josephine Kott Memorial Charitable Trust, the committee developed materials to guide the evaluation process and an operations manual, formed a Board of Governors, registered as an independent non-profit and gained approval as a 501C3 entity. This resulted in the official formation of the Accreditation for Gerontology Education Council. Three schools have gone through the accreditation process. Results of the process will be described and lessons learned will be discussed. Refinement of the process is ongoing. There is a marketing committee that has reached out to additional schools, and new applications are being accepted.

